# Assessment of body mass-related covariates for rifampicin pharmacokinetics in healthy Caucasian volunteers

**DOI:** 10.1007/s00228-024-03697-3

**Published:** 2024-05-09

**Authors:** Muhammad Bilal, Sami Ullah, Ulrich Jaehde, Christina Trueck, Dario Zaremba, Bertil Wachall, Manfred Wargenau, Bernhard Scheidel, Martin H. J. Wiesen, Malaz Gazzaz, Chunli Chen, Sören Büsker, Uwe Fuhr, Max Taubert, Charalambos Dokos

**Affiliations:** 1 Department I of Pharmacology, Center for Pharmacology, Faculty of Medicine and University Hospital Cologne, University of Cologne, Cologne, Germany; 2 Department of Clinical Pharmacy, Institute of Pharmacy, University of Bonn, Bonn, Germany; 3 InfectoPharm Arzneimittel Und Consilium GmbH, 64646 Heppenheim, Germany; 4 M.A.R.C.O. GmbH & Co. KG, Dusseldorf, Germany; 5 ACC GmbH Analytical Clinical Concepts, Leidersbach, Germany; 6 Pharmacology at the Laboratory Diagnostics Centre, Faculty of Medicine, University Hospital Cologne, University of Cologne, Therapeutic Drug Monitoring, Cologne, Germany; 7 Pharmaceutical Practices Department, College of Pharmacy, Umm Al-Qura University, Makkah, Saudi Arabia; 8 Heilongjiang Key Laboratory for Animal Disease Control and Pharmaceutical Development, College of Veterinary Medicine, Northeast Agricultural University, 600 Changjiang Road, Xiangfang District, Harbin, 150030 People’s Republic of China

**Keywords:** Rifampicin, Population pharmacokinetics, Fat-free mass, Body weight, Covariate modeling

## Abstract

**Purpose:**

Currently, body weight-based dosing of rifampicin is recommended. But lately, fat-free mass (FFM) was reported to be superior to body weight (BW). The present evaluation aimed to assess the influence of body mass-related covariates on rifampicin’s pharmacokinetics (PK) parameters in more detail using non-linear mixed effects modeling (NLMEM).

**Methods:**

Twenty-four healthy Caucasian volunteers were enrolled in a bioequivalence study, each receiving a test and a reference tablet of 600 mg of rifampicin separated by a wash-out period of at least 9 days. Monolix version 2023R1 was used for NLMEM. Monte Carlo simulations (MCS) were performed to visualize the relationship of body size descriptors to the exposure to rifampicin.

**Results:**

A one-compartment model with nonlinear (Michaelis–Menten) elimination and zero-order absorption kinetics with a lag time best described the data. The covariate model including fat-free mass (FFM) on volume of distribution (V/F) and on maximum elimination rate (Vmax/F) lowered the objective function value (OFV) by 56.4. The second-best covariate model of sex on V/F and Vmax/F and BW on V/F reduced the OFV by 51.2. The decrease in unexplained inter-individual variability on Vmax/F in both covariate models was similar. For a given dose, MCS showed lower exposure to rifampicin with higher FFM and accordingly in males compared to females with the same BW and body height.

**Conclusion:**

Our results indicate that beyond BW, body composition as reflected by FFM could also be relevant for optimized dosing of rifampicin. This assumption needs to be studied further in patients treated with rifampicin.

**Supplementary Information:**

The online version contains supplementary material available at 10.1007/s00228-024-03697-3.

## Introduction

Tuberculosis (TB) is still the leading cause of death in infectious diseases [1]. According to the World Health Organization (WHO), around 1.6 million people died of an estimated 10.6 million cases from TB in 2021, reflecting an increase of 4.5% from 2020 [2]. In addition, the COVID-19 pandemic has further compromised TB control programs [3].

Rifampicin remains a key anti-TB drug since its introduction in 1968. Rifampicin inhibits DNA-dependent RNA polymerase in *Mycobacterium tuberculosis* and suppresses RNA synthesis by binding to the β-subunit of the enzyme, leading to cell death. Moreover, it treats leprosy and is effective against Gram-positive cocci, including methicillin-resistant staphylococci [4–6].

Rifampicin is readily absorbed from an empty stomach and attains maximum plasma concentrations of approximately 10 mg/L within 2 h following a single dose of 600 mg [7]. Oral absorption of rifampicin is slower when administered with food [8]. The drug is highly lipophilic, and approximately 86 to 89% is bound to plasma proteins [9, 10]. Rifampicin is quickly distributed throughout the bodily fluids, with around 5% of plasma concentrations reaching cerebrospinal fluid [1]. Plasma elimination half-life is approximately 3 to 4 h but decreases to 1 to 2 h after multiple administrations due to massive auto-induction [11]. Both rifampicin and its major metabolite, desacetylrifampicin, are primarily excreted in bile and removed in feces. Up to 30% of the administered dose is renally excreted, and only about 7% of a dose is excreted unchanged in urine [12, 13]. A greater than proportional increase in exposure in plasma is seen when the dose of rifampicin is increased (non-linear pharmacokinetics) [14]. A reduction in the exposure of concomitantly consumed medicines is frequently seen as a result of rifampicin’s extensive induction of various phase I and II metabolic enzymes and drug transporter proteins [1]. Significant induction occurs within several doses after initiating rifampicin therapy, reaches full extent in about 1 week, and disappears within about 2 weeks after discontinuation [15].

The antibacterial effect of rifampicin in patients was formerly thought to be related to *C*_max_/minimum inhibitory concentration (MIC), but recent preclinical investigations have shown that the area under time concentration–time curve (AUC)/MIC is better correlated with the reduction of bacterial counts [16].

It is standard practice to adjust rifampicin doses to total body weight (BW) with 10 mg/kg as the target dose [17]. Lately, fat-free body mass (FFM) was reported to be a better predictor than BW in explaining inter-individual variability of rifampicin exposure, in particular with higher doses where greater variability is expected [18, 19]. Among other possible reasons, increased hepatic metabolism related to higher body size in males was discussed to explain the higher rifampicin clearance [20]. While potential sex differences are more relevant for patients with chronic dosing, assessing such differences in healthy volunteers with a single dose and in the absence of metabolic auto-induction might help understand the background of such an effect. In the present evaluation, population (Pop) PK modeling of rifampicin was applied to data from healthy Caucasian subjects to further assess the variability of PK parameters of rifampicin and to identify the optimal body mass-related predictors of PK parameters.

### Methods

#### Subjects and method

The data were obtained from a phase I/IV randomized, cross-over, open-label bioequivalence study (EUDRACT-No: 2017–004418-24). The study was approved by the Ethics Committee of the Medical Faculty of the University of Cologne (18–006) and carried out in complete agreement with the pertinent version of the Declaration of Helsinki and all other relevant regulations. All volunteers provided written informed consent before participation in the study.

#### Study design

The study was carried out with twenty-five healthy Caucasian volunteers, with one drop-out before the first drug administration. All other volunteers completed the study, and pharmacokinetic and safety data were available in 24 individuals (11 men/13 women). Volunteers had to be between 18 and 85 years old and have a body mass index (BMI) between 18.5 and 30 kg/m^2^. The subjects were deemed fit for the study after extensive standard pre-study screening (medical history, physical examination, vital signs, laboratory tests, electrocardiography, etc.). Main exclusion criteria included hypersensitivity to rifampicin or any of the excipients of the preparations, any relevant clinical abnormality, smoking, chronic or acute medication, extensive ethanol consumption (> 28 g per day for males, > 14 g per day for female subjects), special dietary requirements, and history of substance addiction. Subjects had to abstain from alcohol, methylxanthine-containing beverages, orange juice, apple juice, and grapefruit products, and from extreme physical activities starting 72 h before drug administration. Pregnant and lactating women were also excluded. Participants were randomly allocated to one of the two sequences of the study, each receiving a single dose of either the test or the reference tablet of 600 mg rifampicin first and the alternate treatment after a wash-out period of at least 9 days. Test preparation was a novel rifampicin 600 mg tablet manufactured by InfectoPharm Arzneimittel und Consilium GmbH, Heppenheim, Germany, while reference preparation was a single oral dose of 600 mg tablet (EREMFAT®) manufactured by RIEMSER Pharma GmbH, Frankfurt am Main, Germany.

#### Blood sampling

Blood samples were taken using an indwelling intravenous cannula inserted into a forearm vein. For each PK sample, up to 5 ml of blood was collected in sodium heparinized tubes at predose and 0.16, 0.33, 0.5, 0.75, 1, 1.25, 1.5, 1.75, 2, 2.25, 2.5, 3, 3.5, 4, 6, 9, 12, 16, and 24 h after drug administration. Within 30 min after withdrawal, blood samples were centrifuged at 4 °C at 1992 g for 10 min. After that, the plasma samples were stored at ≤ −70 °C until measurement.

#### Bioanalysis

The quantification of rifampicin was carried out by using a validated liquid chromatography-tandem mass spectrometry (LC–MS/MS) method [21–23]. This process was performed by Analytical Clinical Concepts GmbH, Leidersbach, Germany, and adhered to both EMA and FDA guidelines on bioanalysis. A Shimadzu liquid chromatography system (LC-20AD Pump, Duisburg, Germany) was used for separation. The Analyst® Software version 1.6.2 (AB Sciex, Concord, Canada) was used for data acquisition, peak integration, and quantification of analytes. Rifampicin was obtained from Sigma-Aldrich Chemie GmbH, Taufkirchen, Germany, and rifampicin (rifampicin-d_8_) internal standard (IS) was obtained from Alsachim, Strasbourg, France. 200 µL of plasma was mixed with 500 µL of methanol, 20 µL of ascorbic acid (0.5 mg/L), and 20 µL of the internal standard (rifampicin-d_8_: 100 µg/mL). After shaking the mixture at a speed of 3000 min^−1^, it was centrifuged at 10,500 g for 10 min (4 °C). 50 µL of the supernatant mixed with 400 µL mobile phase was transferred to a reaction vial and stored for 10 min at ≤ 20 °C. The sample was centrifuged for 10 min (4 °C) at 10,500 g, and the supernatant was transferred to an autosampler vial (HTC PAL, CTC Analytics AG, Zwingen, Switzerland). In the LC–MS/MS system, 10 µL was injected. Analytes were separated using a Kinetex® C_18_ chromatographic column (50 × 4.6 mm internal diameter, Phenomenex, Aschaffenburg, Germany) with a pre-column (4 × 3 mm internal diameter, Phenomenex, Aschaffenburg, Germany) and detected using an AB Sciex 2000 (Concord, Canada) mass spectrometer equipped with electrospray ionization source (TurbolonSpray®). The chromatographic separation was achieved by isocratic elution at a flow rate of 0.65 mL/min. The mobile phase consisted of 600 mL ammonium formate (2 mM), 1400 mL methanol, and 2 mL formic acid. The ion spray voltage was 4000 V, and the temperature was set to 400 °C. Ions [M + H]^+^ were detected in multiple reaction monitoring modes using the transitions of *m*/*z* 823.4 → 791.4 for rifampicin and 831.4 → 799.3 for IS, respectively. The column temperature was 25 °C. The linear calibration curve for rifampicin ranged between 100 and 50,000 ng/mL (*r* > 0.9976). The lower limit of quantification (LLOQ) was 100 ng/mL. Stability investigations during method validation showed that rifampicin was stable in plasma at room temperature for at least 6 h and during three thaw/freeze cycles (between ≤ −70 °C and room temperature). For the entire calibration range, accuracy given as a relative deviation of the mean from the nominal value was between −1.0 and 10.7%. The precision expressed in CV was ≤ 8.1% for intra-day and inter-day measurements.

#### Population PK analysis

**Fig. 1:**
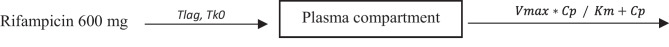
Proposed structural model. Tlag, lag time; Tk0; zero-order process;V_max_, maximum elimination rate; *K*_*m*_, Michaelis–Menten constant; Cp, plasma concentration

Monolix software version 2023R1 (Lixoft®, Antony, France) was used for non-linear mixed effect modeling [24]. The data were fitted using one and two-compartment models with linear and non-linear (Michaelis–Menten) elimination (see Fig. 1). Various absorption models were evaluated, including zero and first order, with and without lag time, and/or with transit compartments. In all models tested, elimination was assumed to take place from the central plasma compartment. The data below the limit of quantification (BQL) was defined as interval-censored at the limit of quantification, 0.1 mg/L [25]. The stochastic approximation expectation–maximization algorithm in Monolix includes simulations of the left-censored data in a right-truncated Gaussian distribution [26]. This is similar to the M4 method implemented in NONMEM to handle BQL data points [27]. Corrected Bayesian Information Criterion (BICc) was used to select non-nested models, and models with the lowest values of BICc were considered superior [28]. Inter-individual variability (IIV) was tested empirically on all PK parameters and was assumed to be log-normally distributed. The two periods were assumed to be two separate occasions, and inter-occasion variability (IOV) was tested empirically on all PK parameters. The correlation between random effects was also investigated, and a strong correlation, i.e., lowering the BICc value by more than 2 points in the non-nested models, was added to the model. To describe the residual variability, constant, proportional, and combined error models were assessed.

#### Covariate analysis

In a prior non-compartmental analysis of this study, it was confirmed that both rifampicin preparations were bioequivalent (data not shown), which allowed us to pool the data for the present analysis. Using the base population pharmacokinetic model, the potential effect of the identity of the rifampicin preparation on rifampicin PK parameters was evaluated as a covariate, along with age, sex, BW, BH (body height), body surface area (BSA), BMI, and FFM. Continuous covariates were modeled using power models normalized by weighted means, i.e., the average of the individual covariate values weighted by the number of observations per individual. Continuous covariates were modeled as shown in Eq. 1, where *PKi* is a PK parameter in the ith subject, PKpop is the population parameter estimation, β is the estimated coefficient of the covariate effect, COV*i* is the value of the covariate for subject *i*, and sex as a categorical covariate was modeled using a linear model where females were taken as reference. Subject characteristics used for covariate model development are given in Table 1. BSA was derived using the Mosteller formula [29], and FFM was calculated from BW and BMI for both males and females, as shown in Eqs. 2 and 3, respectively [30]. Notably, the ranges of FFM for females and males in our study population do not overlap (Table 1). Physiological plausibility and statistical significance, i.e., a reduction in objective value function (OFV) with a decrease of 3.84 (*P* < 0.05) for forward inclusion and an increase in the OFV of 10.8 (*P* < 0.01) for backward elimination [31], usual diagnostic plots (GOF plots), and visual predicted check (VPC), were the basis of selection of the final covariate model. VPC was plotted by simulating 1000 virtual subjects to compare observed data with model-based simulated data to assess the adequate predictive ability of the models. A nonparametric bootstrap analysis (1000 samples) was performed in R using the bootmlx function from Rsmlx (R speaks Monolix, version 2023.1.1) package.

**Table 1:** Subject characteristics used for covariate model development

**Demographics**	**Median (range)**
Sex (male/female)	11/13
Female	
Body weight (kg)	63.8 (48.5–73.1)
Body height (m)	1.65 (1.55–1.76)
FFM (kg)	39.2 (33.7–44.8)
BMI (kg/m^2^)	23.09 (18.7–26.7)
Age (years)	37.0 (21.0–58.0)
BSA (m^2^)	1.66 (1.47–1.85)
Male	
Body weight (kg)	82.9 (57.8–91.0)
Body height (m)	1.81 (1.63–1.89)
FFM (kg)	63 (47.09–68.9)
BMI (kg/m^2^)	24.7 (20.1–29.7)
Age (years)	43.0 (22.0–64.0)
BSA (m^2^)	2.05 (1.62–2.18)

*BMI* body mass index, *FFM* fat-free mass, *kg* kilogram, *m* meter, *m*^*2*^ meter square, *BSA* body surface area

1 $${{{PK}}}_{i}={{{PK}}}_{{{pop}}}*{\left(\frac{{{{COV}}}_{i}}{{{{COV}}i}_{(\mathrm{weighted\; mean})}}\right)}^{\beta }$$

2 $$\mathrm{FFM }\left({{male}}\right)=\frac{9.27*{10}^{3}*{{BW}}}{6.68*{10}^{3}+216*{{BMI}}}$$

3 $$\mathrm{FFM }\left({{female}}\right)=\frac{9.27*{10}^{3}*{{BW}}}{8.78*{10}^{3}+244*{{BMI}}}$$

#### Monte Carlo simulations

Monte Carlo simulations (MCS) were performed for the base model and FFM covariate model only to explore the effect of FFM on exposure to rifampicin. Using the mrgsolve package version 1.0.6 in R, 10,000 virtual subjects were simulated for a single oral dose of 600 mg rifampicin [32].

## Results

### Population pharmacokinetic model

A total of 912 concentrations (median 8.19 (range 0.1 to 31.2) mg/L) obtained from 24 subjects were used for model building, of which 99 observations (10.8%) were BQL. The subject’s median age and body weight were 39.5 years and 68 kg, respectively (Table 1). The PK data of rifampicin in our study was best described by a one-compartment model and zero-order absorption with lag time and nonlinear (Michaelis–Menten) elimination (Fig. 1). A comparison of different base models is given in Table 2. Random effects were applied to describe IIV on the volume of distribution (V/F), maximum elimination rate (Vmax/F), and IOV on lag time (Tlag), zero-order absorption rate (Tk0), V/F, and Vmax/F including a correlation between IOV of Tlag and Tk0. Point estimates of the base model are given in Table 3. A combined additive and proportional error model best explained the residual unexplained variability. The model code and individual fits of all subjects of the base model are shown in Supplementary material.

**Table 2:** Comparisons of different base models with zero- and first-order absorption with or without delay and transit compartments, one or two or three distribution compartments, and linear and non-linear elimination

**Nr:**	**Delay**	**Absorption**	**Distribution**	**Elimination**	**BICc**	∆**BICc**	**OFV**	∆**OFV**
1	Lag time	Zero-order	One cmt	MM	3046	Final base model	2972	Final base model
2	Lag time	First-order	One cmt	MM	3159	113	3083	111
3	Lag time	Zero-order	One cmt	Linear	3180	134	3113	141
4	Lag time	First-order	One cmt	Linear	3270	224	3208	236
5	Transit cmt	First-order	One cmt	MM	3688	642	3622	650
6	Transit cmt	First-order	One cmt	Linear	3761	715	3702	730
7	No delay	Zero-order	One cmt	MM	4636	1590	4591	1619
8	No delay	Zero-order	One cmt	Linear	4696	1650	4655	1683
9	No delay	First-order	One cmt	Linear	4757	1711	4713	1741
10	No delay	First-order	One cmt	MM	4765	1719	4714	1742

*BICc* Corrected Bayesian Information criteria, *OFV* objective function value, *cmt* compartment, *MM* Michaelis–Menten, ***∆****BICc* change in BICc value, ***∆****OFV* change in OFV

**Table 3:** Parameter estimates of base and covariate models and bootstrap medians with respective 95% confidence intervals of the sex + body weight and FFM covariate models

	Base model	Sex + BW covariate model		FFM covariate model	
OFV	2972.77	2921.53		2916.33	
Parameter	Estimates (RSE %)	Estimates (RSE %)	Bootstrap median (95% CI)	Estimates (RSE %)	Bootstrap median (95% CI)
**Fixed effects**					
Tlag (h)	0.340 (5.52)	0.340 (5.49)	0.338 (0.300–0.382)	0.340 (5.53)	0.337 (0.301–0.382)
Tk0 (h)	0.460 (9.17)	0.470 (9.02)	0.466 (0.382–0.561)	0.470 (8.99)	0.463 (0.386–0.561)
V/F (L)	36.2 (5.28)	33.2 (3.11)	33.1 (31.0–36.4)	36.2 (2.21)	36.1 (34.5–38.2)
Vmax/F (mg/h)	191 (7.28)	157 (6.70)	154 (134–175)	190 (4.39)	188 (167–209)
*K*_*m*_ (mg/L)	20.4 (5.45)	20.2 (4.70)	19.7 (16.6–22.5)	20.1 (2.44)	19.8 (17.3–22.9)
**Covariate effect**					
β sex on V/F_sex+BW_	-	0.190 (24.6)	0.190 (0.079–0.280)		
β BW on V/F_sex+BW_		1.00 (Fixed)	1.00		
β sex on Vmax/F_sex+BW_		0.420 (20.3)	0.417 (0.252–0.417)		
β FFM on V/F_FFM_	-	-	-	1.00 (fixed)	1.00
FFM on Vmax/F_FFM_		-	-	0.750 (fixed)	0.750
**Random effects and correlation**					
IIV V/F (CV %)	24.4 (17.4)	-	-	-	-
IIV Vmax/F (CV %)	39.0 (15.7)	19.7 (16.7)	20.2 (13.9–25.6)	18.5 (16.9)	19.6 (13.9–23.9)
IOV Tlag (CV %)	39.1 (10.5)	38.8 (10.5)	44.5 (28.0–63.7)	39.0 (10.6)	44.8 (27.3–63.3)
IOV Tk0 (CV %)	66.9 (11.3)	65.7 (11.1)	80.7 (58.7–106.2)	65.5 (11.2)	79.4 (56.8–105)
IOV V (CV %)	13.5 (15.9)	15.4 (10.9)	15.9 (9.52–23.6)	14.9 (10.9)	15.3 (9.63–22.8)
IOV Vmax (CV %)	9.38 (16.4)	9.32 (16.4)	9.52 (6.93–12.4)	9.39 (16.4)	9.63 (7.14–12.2)
Corr. IOV Tlag & IOV Tk0	0.390 (33.7)	0.420 (31.0)	0.439 (0.051–0.659)	0.410 (31.6)	0.425 (0.079–0.670)
**Error model parameters**					
Additive residual error (mg/L)	0.061 (8.18)	0.060 (7.85)	0.060 (0.049–0.070)	0.060 (7.69)	0.060 (0.048–0.069)
Proportional residual error (%)	11.0 (3.31)	11.0 (3.33)	10.8 (9.70–12.0)	11.0 (3.31)	10.8 (9.80–11.9)

*OFV* Objective function value, *BW* body weight, *RSE* relative standard error, *CV* coefficient of variation, *Tk0* zero-order absorption, *Tlag* lag time, *V/F* volume of distribution, *Vmax/F* maximum elimination rate, *K*_*m*_ Michaelis–Menten constant, *bio* bioavailability, *FFM* fat-free mass, *Sex*_*V/F*_ effect of sex on the volume of distribution, *BW*_*V/F*_ effect of body weight on volume of distribution, *FFM*_*V/F*_ effect of fat-free mass on volume of distribution, *IIV* inter-individual variability, *IOV* inter-occasion variability, *Corr* correlation, *CI* confidence interval, *β* estimated coefficient of the covariate effect

### Covariate modeling

Covariates tested on PK parameters were significant on V/F and Vmax/F (Table 4). The best covariate model included FFM only, followed by the sex + BW model. The covariate model with FFM on both V/F and Vmax/F decreased the objective function value (OFV) by 56.4 points compared with the base model. Adding sex as a separate covariate in addition to FFM did not improve the model further. An alternative covariate model including sex on both V/F as well as Vmax/F and BW on V/F lowered the OFV by 51.2 points. The residual IIV on Vmax/F in the base model was 39.0 (CV (coefficient of variation) %), which was lowered to 18.5 and 19.7 for FFM and sex + BW covariate models, respectively. Estimating power parameters empirically did not result in a statistically significant improvement in the covariate models. Replacing BW by BSA in this model lowered the OFV by 3.24 points from the sex + BW model however failed to meet the backward deletion criteria. Body height only was also significant on V/F and Vmax/F but to a lower extent than the covariate models mentioned above, i.e., lowering OFV by 42.9 points. When tested on Tlag and V/F, BMI was significant and lowered OFV by 4.02 and 5.11, respectively. However, it did not meet the backward elimination criteria. Including the covariates explained most of the variability of V/F, making IIV on V/F non-significant. The point estimates of the sex + BW and the FFM covariate models are shown in Table 3. The identity of the preparation (test or reference) had no significant effect on any of the parameters.

**Table 4:** Summary of covariate models with change in objective function value

Nr:	Covariate model	∆OFV
1	BW on V/F & Vmax/F	- 37.1
2	Sex on V/F & Vmax/F	- 38.4
3	BH on V/F & Vmax/F	- 42.9
4	Sex on Vmax/F and BSA on V/F	- 47.07
5	BSA on V/F & Vmax/F	- 48.2
6	Sex on V/F & Vmax/F and BW on V/F	- 51.2
7	FFM on V/F & Vmax/F	- 56.3

*∆OFV* change in objective function value, *BH* body height, *BW* body weight, *FFM* fat-free mass, *V/F* volume of distribution, *Vmax/F* maximum elimination rate

### Model evaluation

**Fig. 2:**
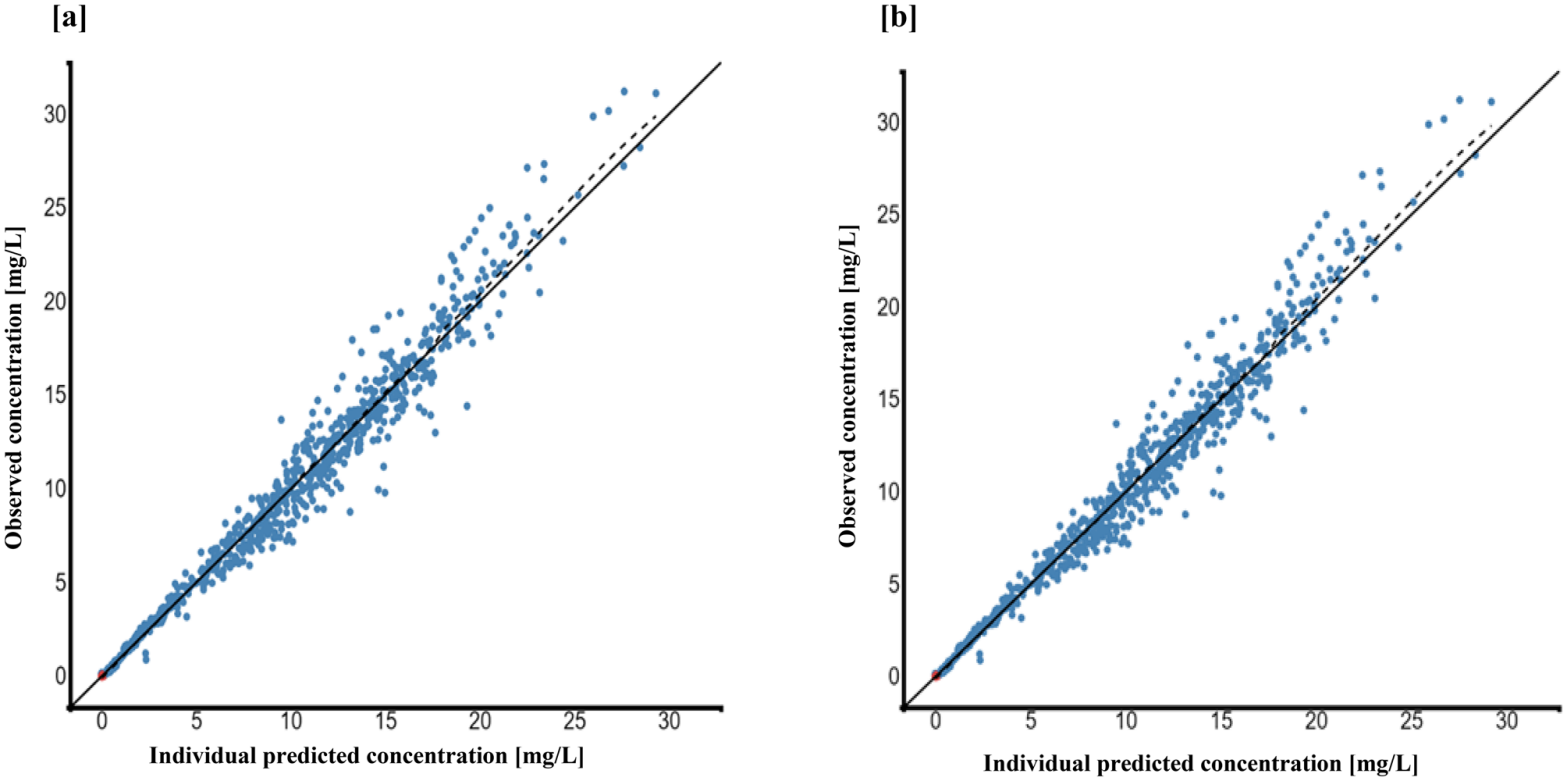
Individual predictions vs observations of **a** sex + BW and **b** FFM covariate models. Solid blue dots represent observed concentration, and solid red dots represent data below the limit of quantification (BQL). The black line is the line of unity, and the dotted line represents the spline

**Fig. 3:**
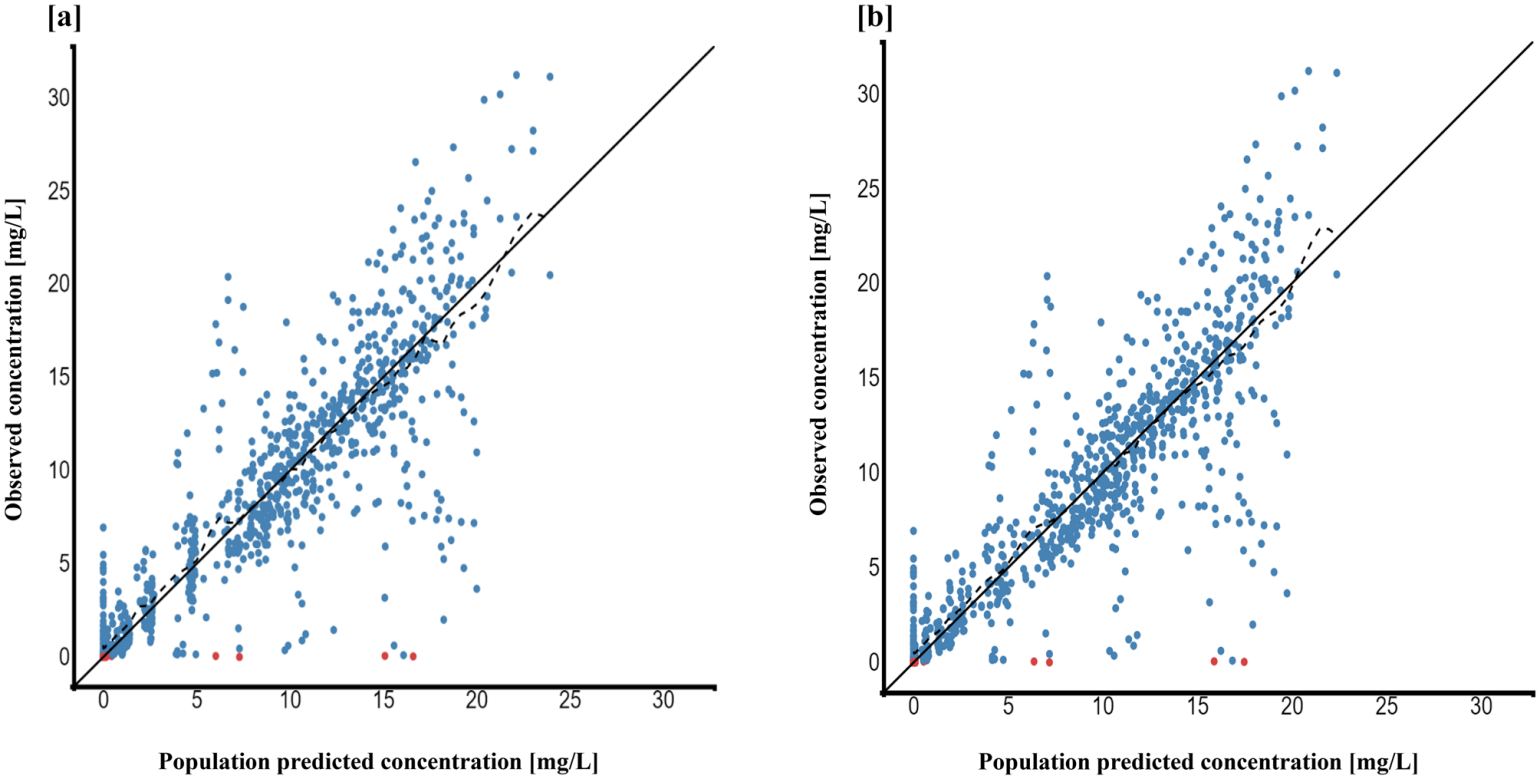
Population predictions vs observations of **a** sex + BW and **b** FFM covariate models. Solid blue dots represent observed concentration, and solid red dots represent BQL data. The black line is the line of unity, and the dotted line represents the spline

**Fig. 4:**
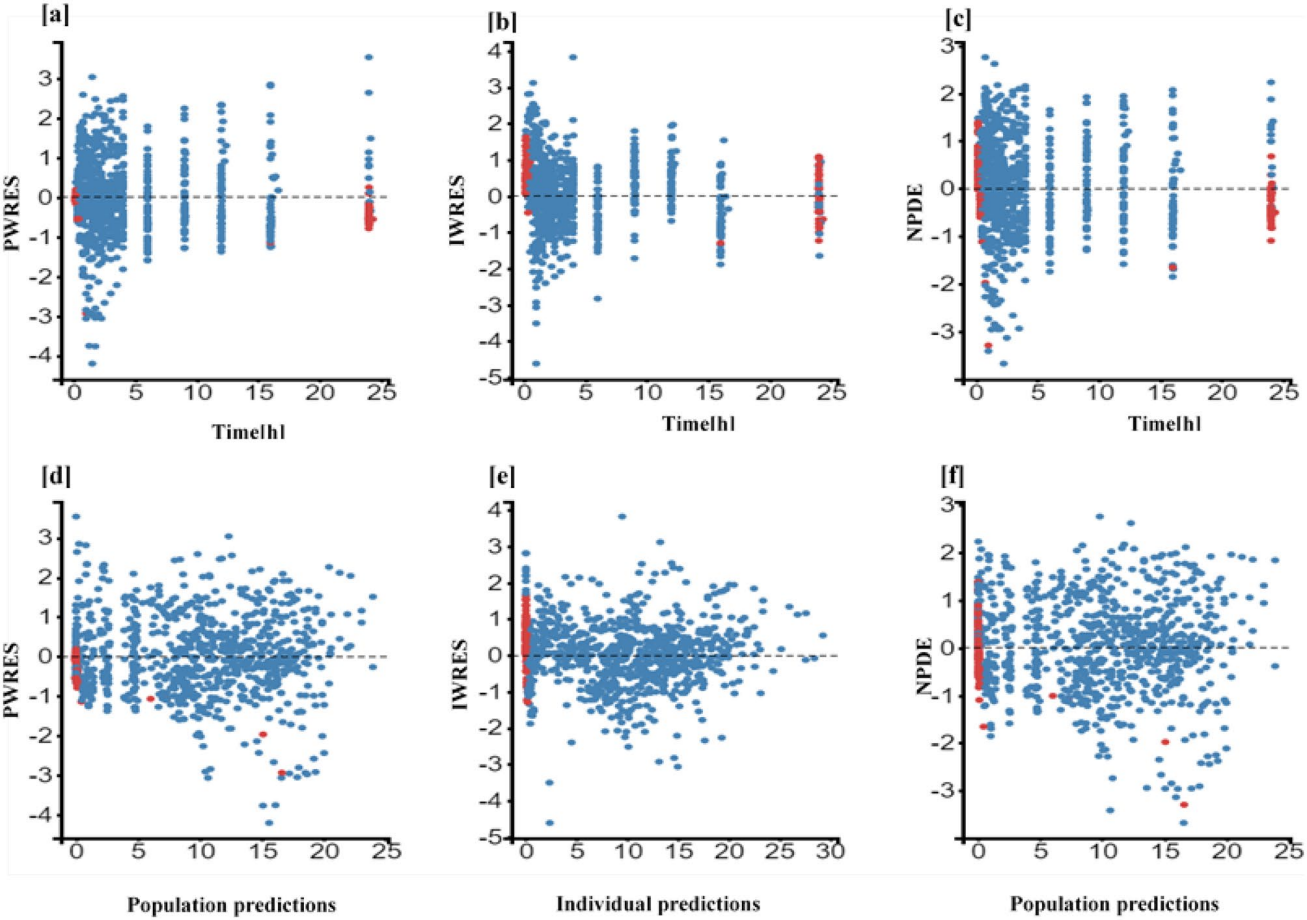
Scatter plots of the residuals of sex + weight covariate model. The dotted line is the mean of the residuals. Solid red dots represent simulated observations below the limit of quantification data. **a** PWRES versus time, **b** IWRES versus time, **c** NPDE versus time, **d** PWRES versus population prediction, **e** IWRES versus individual prediction, and **f** NPDE versus population prediction. PWRES, population-weighted residuals; IWRES, individual weighted residuals; NPDE, normalized prediction distribution errors

**Fig. 5:**
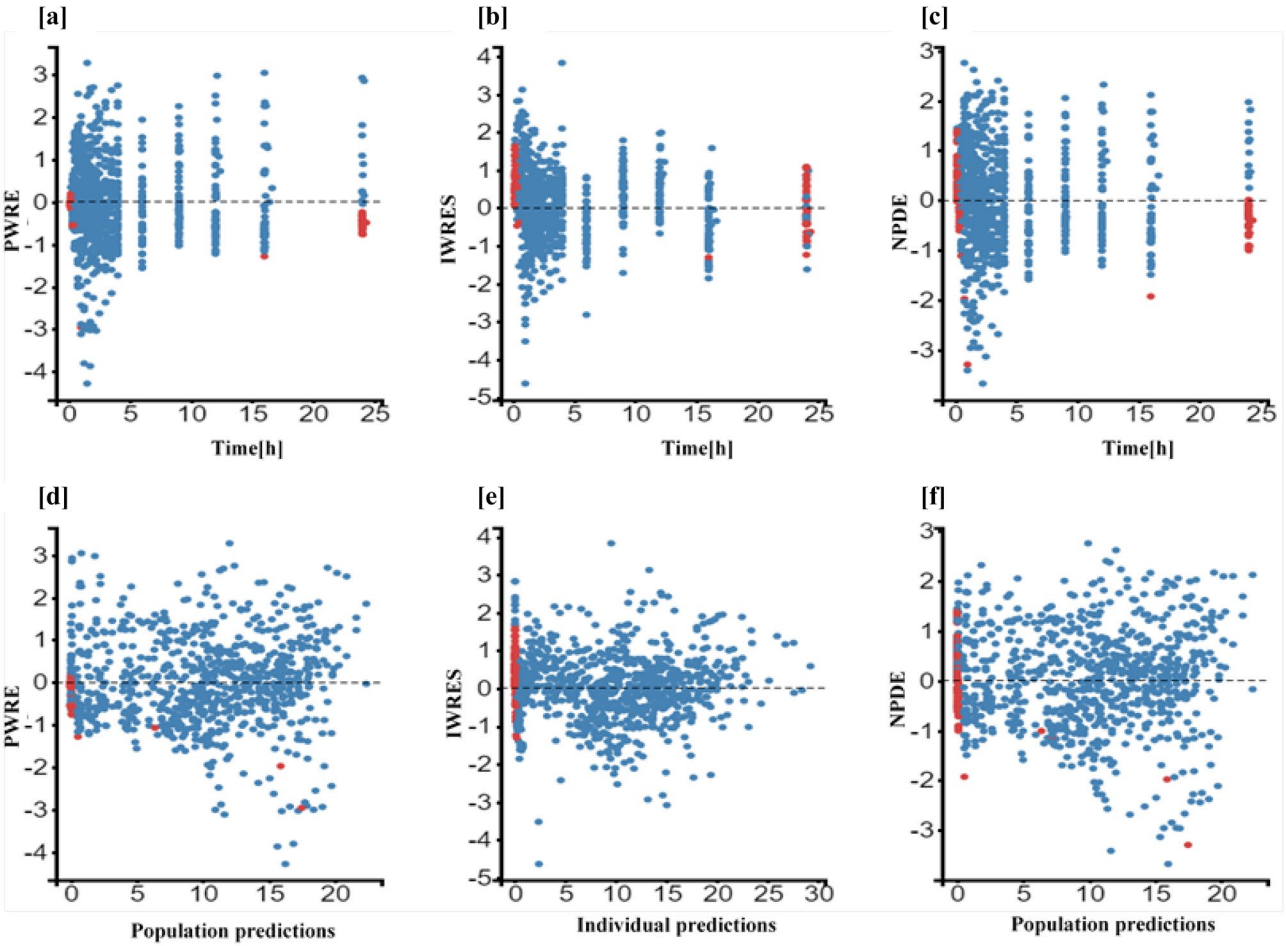
Scatter plots of the residuals of the FFM covariate model. The dotted line is the mean of the residuals. Solid red dots represent simulated observations below the limit of quantification data. **a** PWRES versus time, **b** IWRES versus time, **c** NPDE versus time, **d** PWRES versus population prediction, **e** IWRES versus individual prediction, and **f** NPDE versus population prediction. PWRES, population-weighted residuals; IWRES, individual weighted residuals; NPDE, normalized prediction distribution errors

**Fig. 6:**
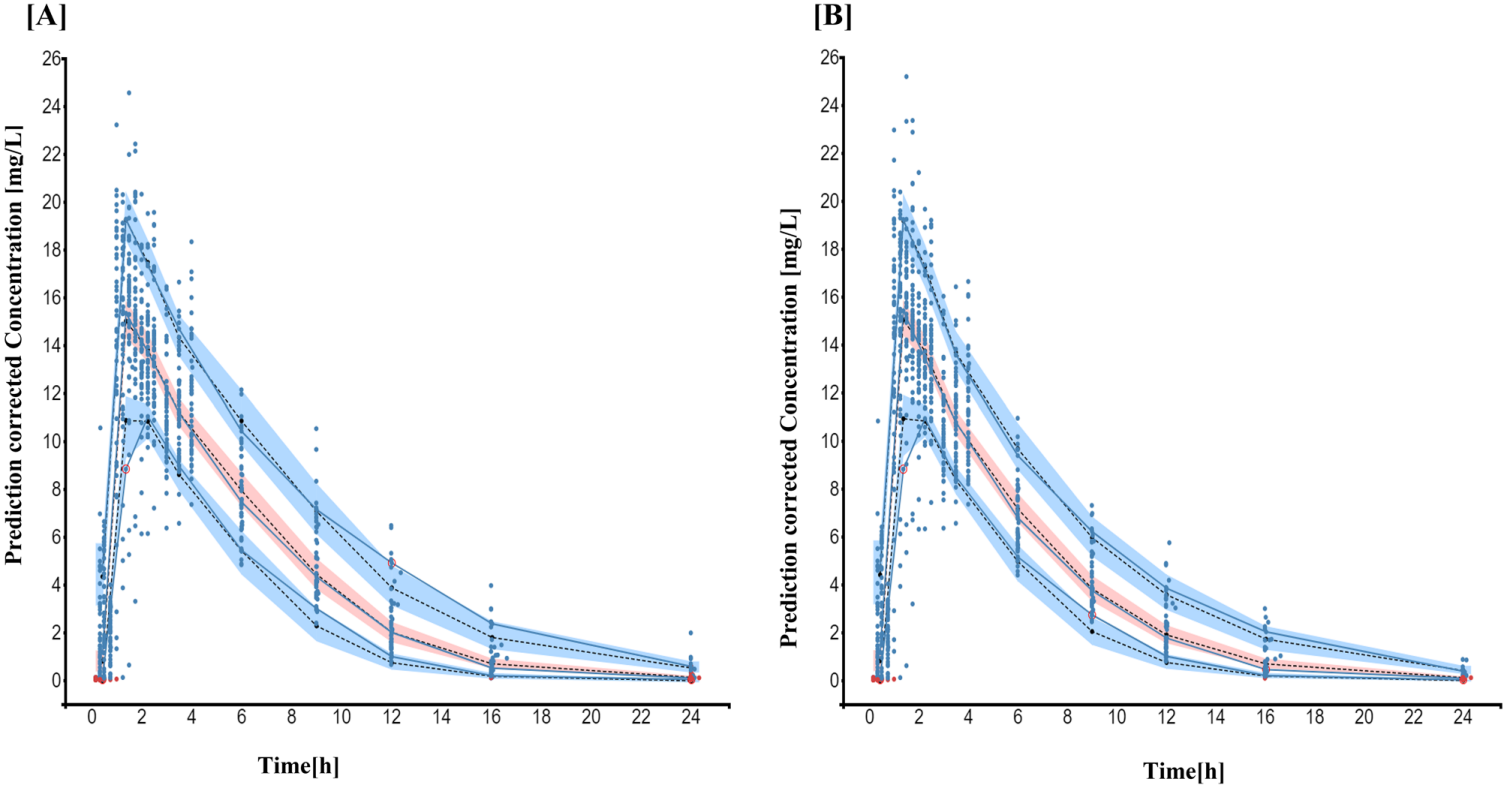
Prediction corrected visual predictive check (*n* = 1000) of **A** sex + BW and **B** FFM covariate model. Solid blue dots represent observed concentrations. Solid red dots represent BQL data. Solid blue lines represent observed concentrations’ median and 10th and 90th percentiles. Shaded areas are the model-predicted 90% confidence intervals of 10th, 50th, and 90th percentiles (lower blue area, red area, upper blue area, respectively). Black dotted lines represent medians of the respective confidence intervals of simulated data

The individual and population prediction plots for sex + BW and FFM covariate models are shown in Figs. 2 and 3, respectively. Observations were uniformly distributed along the identity line for individual and population predictions of both covariate models except for early high concentrations, i.e., above or ~ 25 mg/L in population prediction plots. No systematic over- or under-prediction was evident from plots of residuals (see Figs. 4 and 5). Figure 6 shows the prediction corrected (pc) VPC for both sex + BW and FFM covariate models. The figures show that both the models captured the central trend and variability in the data. A semi-logarithmic pc-VPC is provided in supplementary Fig. 2. All parameter point estimates were within the 95% CI and close to the bootstrap median (Table 3).

### Monte Carlo simulations

**Fig. 7:**
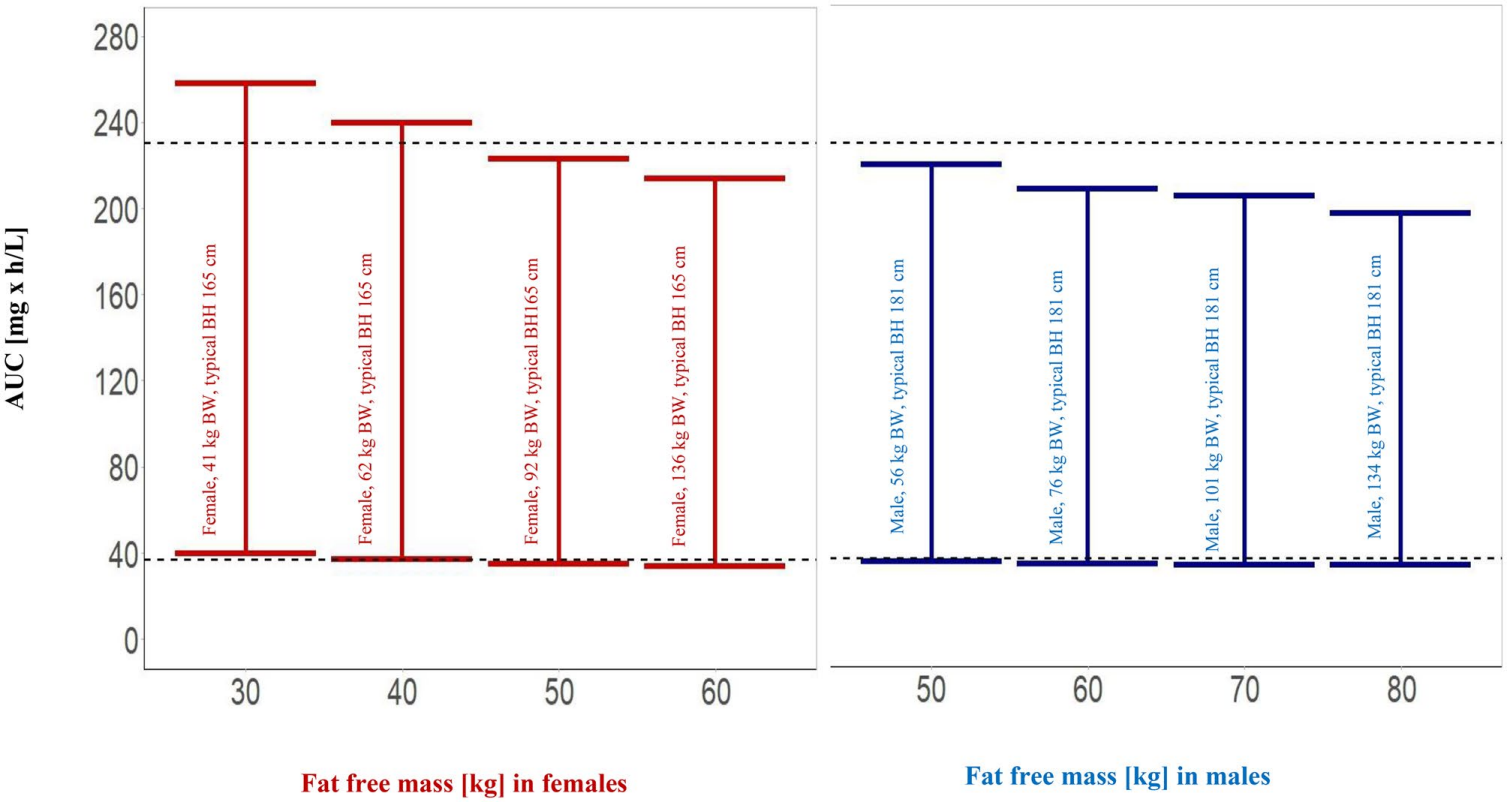
Effect of weight on simulated exposure of rifampicin for a 600 mg oral dose with FFM as a covariate and without covariate effect. Bars represent 5th and 95th percentiles, and dotted black lines represent 5th and 95th percentiles without taking a body size-related covariate into account. AUC, area under the concentration–time curve. Body weight (BW) is given with each FFM value for a male individual with a typical body height (BH) in the study population of 185 cm and a female individual with a typical body height of 165 cm to illustrate the meaning of FFM

Figure 7 illustrates the relationship of FFM to exposure to rifampicin. AUC and FFM ranging from 30 to 60 kg in female individuals and from 50 to 80 kg in male individuals were simulated for the FFM covariate model and the base model without covariates for an oral dose of 600 mg. MCS showed lower overall exposure to rifampicin with higher FFM. For each FFM value used for simulations, biometric characteristics for a male and a female individual with respective typical body height in our population are shown to illustrate the meaning of FFM. Furthermore, the error bars show a significant reduction in variability comparing the model with FFM to the base model, indicating that the covariates explained a relevant extent of random variability.

## Discussion

We developed a population PK model of rifampicin based on a rich blood sampling schedule in healthy subjects. We found that either FFM or a combination (second-best) of body weight and sex explained some of the pharmacokinetic variability better than body weight alone did.

Published PK models of rifampicin vary in terms of absorption, presence of non-linearity, and auto-induction components, all typical characteristics of rifampicin. Most studies reported a one-compartment model with various approaches to describe absorption, including first-order absorption, sequential zero, and first-order absorption with lag time [20, 33–36], or incorporating transit compartments [37–42]. Most studies reported first-order elimination [20, 36, 37, 42–45], while a few investigations reported saturable elimination for rifampicin [19, 39, 41]. A two-compartment model [46, 47] and a three-compartment model have also been reported in the literature for rifampicin [48]. In the present study, one distribution compartment with zero-order absorption and lag time linked with Michaelis–Menten elimination best fits the data.

The identity of the rifampicin preparation in this study did not influence the pharmacokinetic parameters, including those describing drug absorption. A study conducted by Männistö nicely demonstrated that the bioavailability of oral preparations of rifampicin may differ considerably, with liquid preparations achieving much higher bioavailability [49]. It is difficult to predict the bioavailability of immediate release solid oral rifampicin preparation by in vitro dissolution studies, which is mainly attributable to the poor solubility at neutral pH, making rifampicin a BCS (Biopharmaceutics Classification System) class 2 drug [50]. In addition, a more than linear increase of exposure with the dose may contribute to the poor predictability of the bioavailability of rifampicin preparations.

Since the early 1970s, it has been known that rifampicin exposure increases more than linearly with dose [51], with saturable hepatic extraction/saturable biliary excretion being the reported reasons [7, 19, 51]. Several studies have also reported saturable (Michaelis–Menten) elimination of rifampicin [16, 35], which is confirmed by our results. We could not include auto-induction in our model, which is to be expected as only a single dose of rifampicin was administered. In other reports, rifampicin is also reported to follow first-order elimination. However, in the respective population’s PK models, rifampicin is administered along with other anti-TB drugs and/or other medication for comorbidities, and sampling densities may not have been suitable to derive more complex PK models [35, 37, 52].

A typical form of dose individualization is drug dosing based on total body weight. The use of weight-band dosing of rifampicin is well established. BW has been reported to be a significant covariate on clearance and volume of distribution of rifampicin. A decrease of 8% in unexplained IIV using BW as a covariate on volume of distribution had been reported [53], while Schipani et al. reported a reduction of 15.5% in a joint covariate model of weight and age on clearance [45]. However, Susanto et al. reported that weight-band dosing of rifampicin could not reduce between-subject variability in AUC_0–24_ for high doses in adult TB patients. The authors concluded that weight-band dosing of rifampicin does not provide any benefit over flat dosing [54]. Despite body weight-adjusted dosing, previous studies indicated that in comparison to females, males are more likely to have lower plasma rifampicin concentrations﻿ [55]. When tested as a covariate, male gender increased the value of clearance and volume of distribution by 40% and 29%, respectively, in Mexican patients with TB [20]. Medellín-Garibay et al. reported a high volume of distribution and clearance in male individuals compared to females [36]. In one of our models, sex together with body weight were significant covariates and jointly decreased residual IIV on Vmax/F by 19.3 (CV%) (Table 3), while the FFM-based model (in terms of OFV) suggests that the relationship to sex may be the result of different body composition between men and women (see below) Still, despite being related to Vmax/F, none of the significant covariates in our models are causally linked to this parameter and therefore must be regarded as empirical surrogate parameters. Overall, the majority of available data supports that body weight as a descriptor of body size improves the prediction of rifampicin exposure. However, it may not be the most suitable or only useful respective descriptor, as body composition also depending on sex is not taken into account when dosing rifampicin based on body weight.

Indeed, FFM as another body size descriptor in the pharmacokinetic literature performed better than BW + sex or BSA + sex in our evaluation. FFM was derived in 1945 by Rathbun and Pace [56]. Jeremiah et al. reported that FFM is a better size predictor of both clearance and volume of distribution of rifampicin when compared to BW in TB patients coinfected with HIV [39]. A semi-mechanistic model of FFM developed by Janmahasatian et al. [30] by incorporating sex, body weight, and BH was used for FFM covariate modeling (Eqs. 2 and 3). FFM was the most significant covariate of all the covariates tested (Table 4) and decreased residual IIV on Vmax/F by 20.5(CV%) (Table 3). The simulation results in our study, based on the FFM model, illustrate (Fig. 7) the degree to which exposure depends on FFM. According to the differences in FFM, the exposure of rifampicin was higher in females than males and decreased in both sexes with an increase in FFM. Measuring FFM requires experimental procedures that are complex and/or costly, precluding their application in standard clinical practice. The methods used to experimentally assess FFM vary depending on principles such as whole-body counting, bioimpedance, densitometry, dual-energy X-ray absorptiometry, medical imaging, and hydrometry. They differ in their methods and fundamental biological presumptions that are often not applicable to some populations, such as children, the elderly, and those with specific disease states [57]. As a result, models that forecast FFM from quantifiable factors, including body weight and height, are employed in both population pharmacokinetic modeling and clinical practice. It is unclear which of the various procedures to forecast FFM are the most reliable [57]. This uncertainty is a caveat for the use of FFM for individualized dosing, and using covariates that are available without further assumptions such as body weight together with sex has the advantage of easy implementation.

The U.S. Food and Drug Administration recommends BSA scaling for using animal model species data to establish safe starting doses for the first in human clinical studies [58]. Most established BSA formulae are based on variables including body weight and height [59]. In 1987, Mosteller [29] introduced a simplified method of the BSA equation initially proposed by Gehan and George [60] without taking sex differences into account. Sex differences in the pharmacokinetics of drugs have been reported in the literature. FDA identified statistically significant sex differences in about 28% of data sets from bioequivalence trials and suggested that drug exposure difference could exceed 50% [61]. Similarly to FFM, BSA is also a derived parameter based on an individual’s BW and body height. When considered with sex, BSA did not offer a significant advantage over BW and sex only (Table 4). BMI is currently the typical worldwide measure for classifying obesity. In this study and the study conducted by Gao et al., BMI was not a significant covariate on PK parameters of rifampicin [52]. BMI increases with total body weight but cannot distinguish adipose tissue from muscle mass, and its usefulness as a dosage scalar is restricted because patients with a large muscle mass would get the same dose as patients with a large fat mass. Additionally, BMI is not sex-specific, is not derived using data from women, and has not been tested for its ability to predict morbidity in women [56].

This study has a few limitations. It was primarily designed for assessing the bioequivalence of two rifampicin preparations and not for covariate analysis. Furthermore, it was designed as a single-dose study and not a multiple-dose study; thus, autoinduction of rifampicin metabolism could not be considered. On the other hand, identification of a sex effect with single doses avoids a potential bias caused by possible sex differences in autoinduction and thus may help assess individual components in rifampicin pharmacokinetics explaining sex differences. This study only included healthy volunteers from a Caucasian population. Further analysis would be required in TB patients and other populations, and it should include the achievement of pharmacokinetic/pharmacodynamics targets to assess the clinical relevance differences in rifampicin exposure based on body composition.

Based on PK principles, it stands to reason that FFM is the primary biological covariate directly affecting PK in our evaluation, while sex exerts its effect as a covariate indirectly via affecting FFM. The current approach to derive FFM has the disadvantage that it is estimated from sex, body weight, and height only (Eqs. 2 and 3) and thus takes individual body composition to some degree into account while it does not consider fat vs. muscle mass within the male and female groups. Estimated FFM was the best covariate to explain inter-individual variability in PK of rifampicin in healthy volunteers indicating that body composition could also be considered for optimized dosing of rifampicin. The assumption that FFM is preferable to BW confirms previous findings in the African population [39] but needs to be studied further in Caucasian and Asian patients treated with rifampicin.

### Supplementary Information

Below is the link to the electronic supplementary material.

Supplementary file1 (DOCX 2088 KB)
