# Supplementary material for: Assessment of body mass-related covariates for rifampicin pharmacokinetics in healthy Caucasian volunteers
Source: Eur J Clin Pharmacol. 2024 May 9;80(9):1271–83. doi: 10.1007/s00228-024-03697-3 (PMC11303472; doi:10.1007/s00228-024-03697-3)
Supplement: Supplementary file 1 — Supplementary file1 (DOCX 2088 KB) [file 228_2024_3697_MOESM1_ESM.docx]

**Assessment of body mass-related covariates for rifampicin pharmacokinetics in healthy Caucasian volunteers**

Muhammad Bilal^1, 2*^, Sami Ullah^1^, Ulrich Jaehde^2^, Christina Trueck^1^, Dario Zaremba^1^, Bertil Wachall^3^, Manfred Wargenau^4^, Bernhard Scheidel^5^, Martin H. J. Wiesen^6^, Malaz Gazzaz^7^, Chunli Chen^1,8^, Sören Büsker^1^, Uwe Fuhr^1^, Max Taubert^1^, Charalambos Dokos^1^

^1^ Department of Pharmacology, Faculty of Medicine and University Hospital Cologne, University of Cologne, Cologne, Germany

^2^ Department of Clinical Pharmacy, Institute of Pharmacy, University of Bonn, Bonn, Germany

^3^ InfectoPharm Arzneimittel und Consilium GmbH, 64646 Heppenheim, Germany

^4^ M.A.R.C.O. GmbH & Co. KG, Düsseldorf, Germany

^5^ ACC GmbH Analytical Clinical Concepts, Leidersbach, Germany

^6^ Pharmacology at the Laboratory Diagnostics Centre, Therapeutic Drug Monitoring, Faculty of Medicine and University Hospital Cologne, University of Cologne, Cologne, Germany

^7^ Pharmaceutical Practices Department, College of Pharmacy, Umm Al-Qura University, Makkah, Saudi Arabia

^8^ Heilongjiang Key Laboratory for Animal Disease Control and Pharmaceutical Development, College of Veterinary Medicine, Northeast Agricultural University, 600 Changjiang Road, Xiangfang District, Harbin 150030, PR China

**#corresponding author**

Muhammad Bilal

Department I of Pharmacology, Faculty of Medicine and University Hospital Cologne

Center for Pharmacology, University of Cologne

Gleueler Straße 24, 50931, Cologne, Germany

Email: Muhammad.bilal@uk-koeln.de

KEYWORDS

Rifampicin, population pharmacokinetics, fat-free mass, body weight, covariate modelling

**Supplementary Material:**

**Model code of the base model**:

[INDIVIDUAL]

input = {Km_pop, V_pop, Vm_pop, Tlag_pop, Tk0_pop, omega_V, omega_Vm, gamma_Tlag, gamma_Tk0, gamma_V, gamma_Vm, corr2_Tlag_Tk0}

DEFINITION:

Km = {distribution=logNormal, typical=Km_pop, no-variability}

V = {distribution=logNormal, typical=V_pop, varlevel={id, id*occ}, sd={omega_V, gamma_V}}

Vm = {distribution=logNormal, typical=Vm_pop, varlevel={id, id*occ}, sd={omega_Vm, gamma_Vm}}

Tlag = {distribution=logNormal, typical=Tlag_pop, varlevel=id*occ, sd=gamma_Tlag}

Tk0 = {distribution=logNormal, typical=Tk0_pop, varlevel=id*occ, sd=gamma_Tk0}

correlation = {level=id*occ, r(Tlag, Tk0)=corr2_Tlag_Tk0}

[LONGITUDINAL]

input = {a, b}

;;;; Included file 'oral0_1cpt_TlagTk0VVmKm.txt'

DESCRIPTION:

The administration is extravascular with a zero order process (duration Tk0) with a lag time (Tlag).

The PK model has one compartment (volume V) and a Michaelis-Menten elimination (Vm, Km).

input = {Tlag, Tk0, V, Vm, Km}

EQUATION:

odeType = stiff

PK:

; PK model definition

Cc = pkmodel(Tlag, Tk0, V, Vm, Km)

OUTPUT:

output = {Cc}

;;;;

DEFINITION:

DV = {distribution=normal, prediction=Cc, errorModel=combined1(a, b)}

**Observed concentration [mg/L]**

**Time[h]**


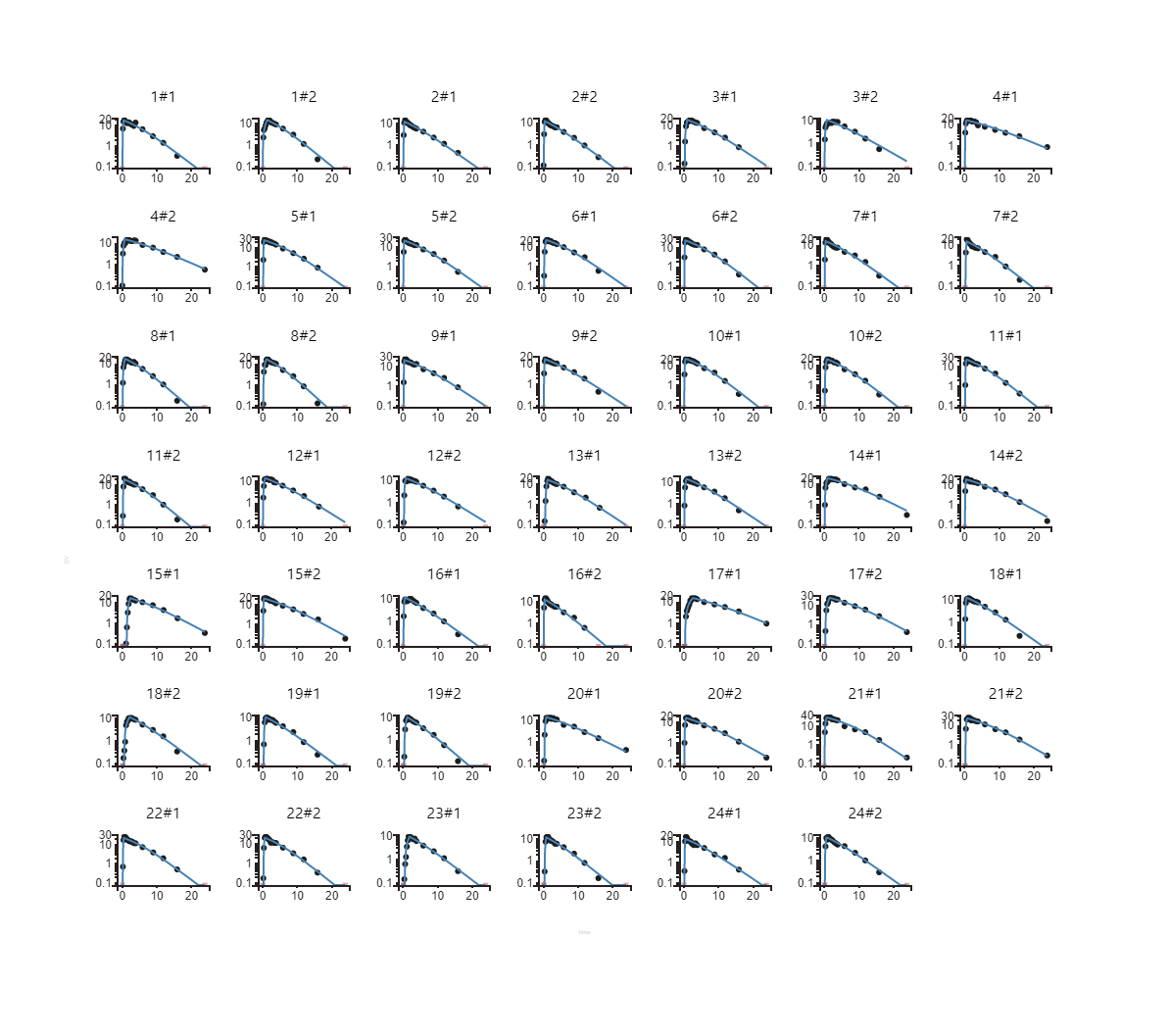


**Supplementary Fig 1.** Individual fits of all the subjects of the base model. #1 and #2 designate test and reference preparations, respectively, and solid dark dots represent observed concentration. Blue lines represent the prediction.

v

**Concentration [log_10_ mg/L]**

**Time[h]**


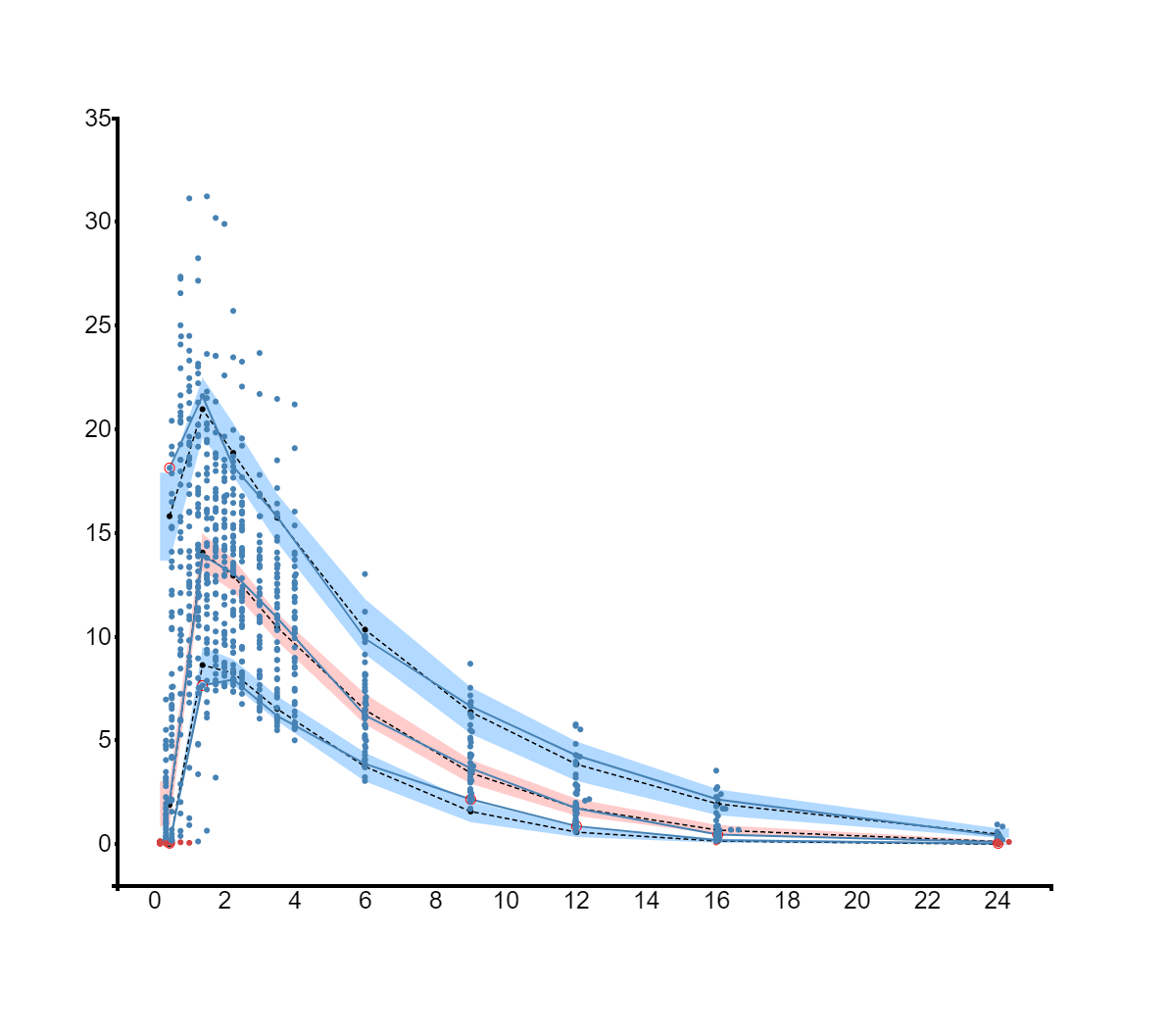


**[A]**

**Concentration [log_10_ mg/L]**

**Time[h]**


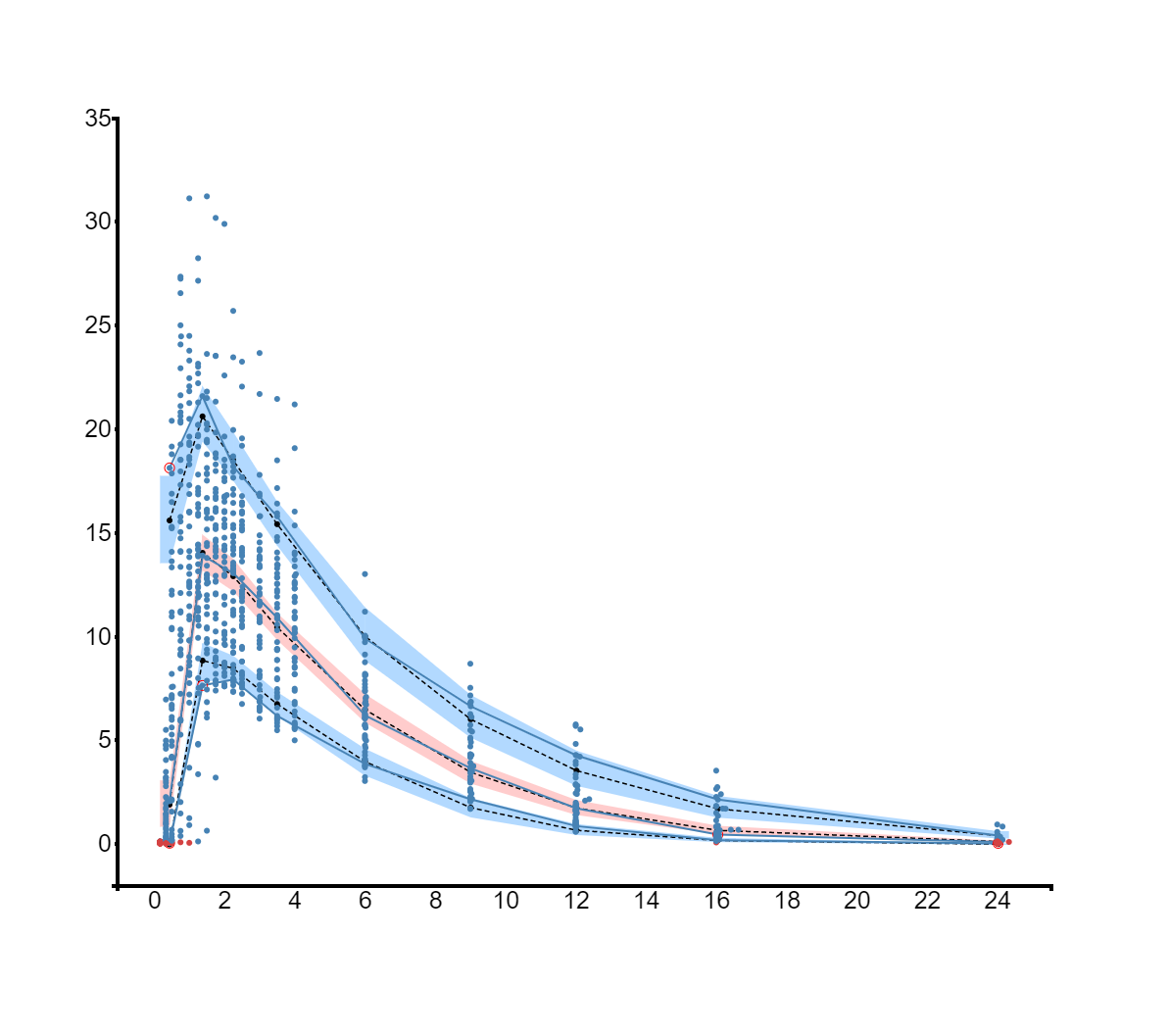


**[B]**

**[A]**

**Supplementary Fig 2.** Prediciton corrected visual predictive check (n = 1000) of [A] sex + BW and [B] FFM covariate model. Solid blue dots represent observed concentrations. Solid red dots represent BQL data. Solid blue lines represent median, 10^th,^ and 90^th^ percentiles of observed concentrations. Shaded areas are the model-predicted 90% confidence intervals of 10^th^, 50^th^, and 90^th^ percentiles (lower blue area, red area, upper blue area, respectively). Black dotted lines represent medians of the respective confidence intervals of simulated data.

**Prediction corrected Concentration [log_10_ mg/L]**

**[A]**

**Time[h]**


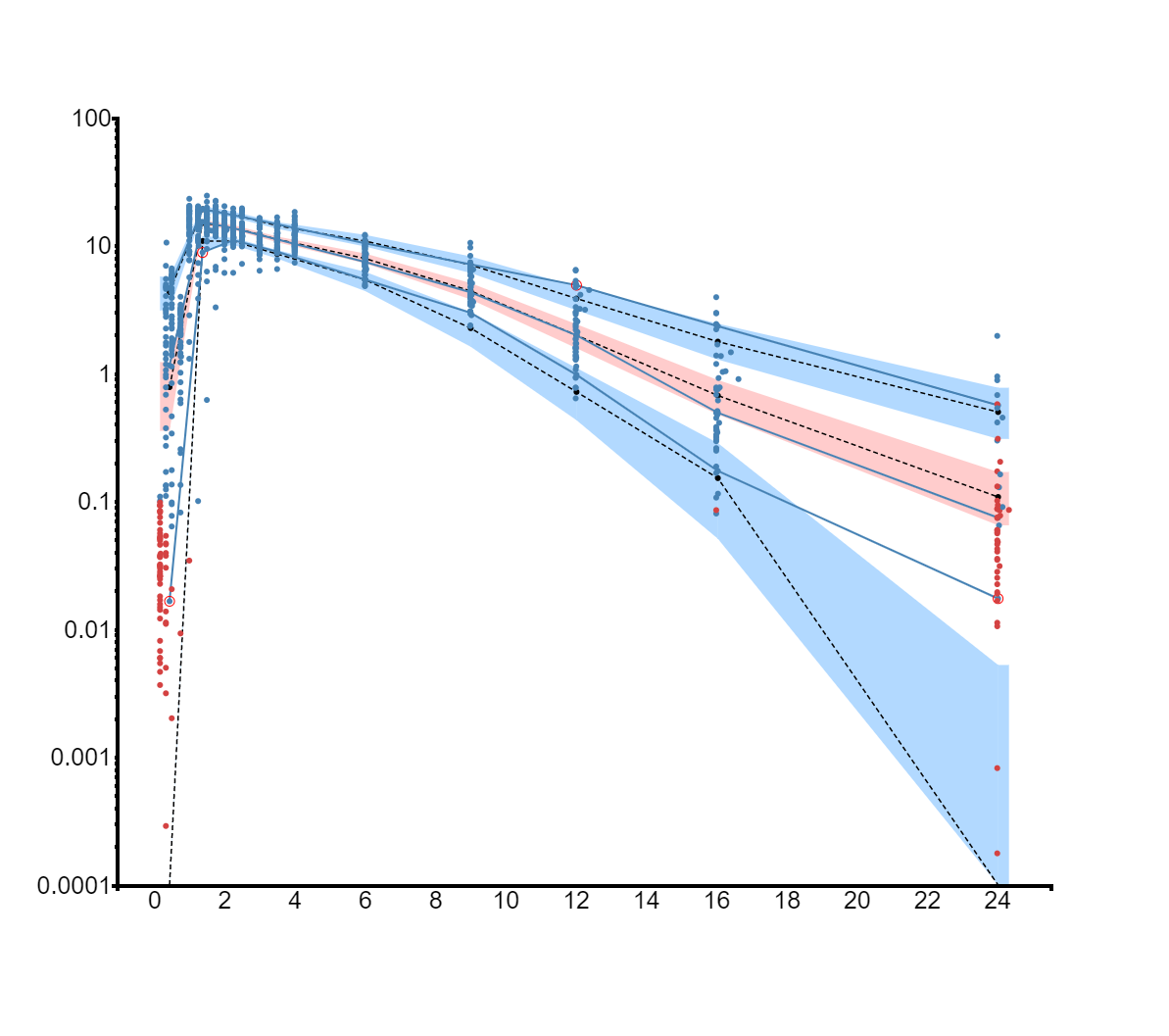


**Prediction corrected Concentration [log_10_ mg/L]**

**Time[h]**

**[B]**


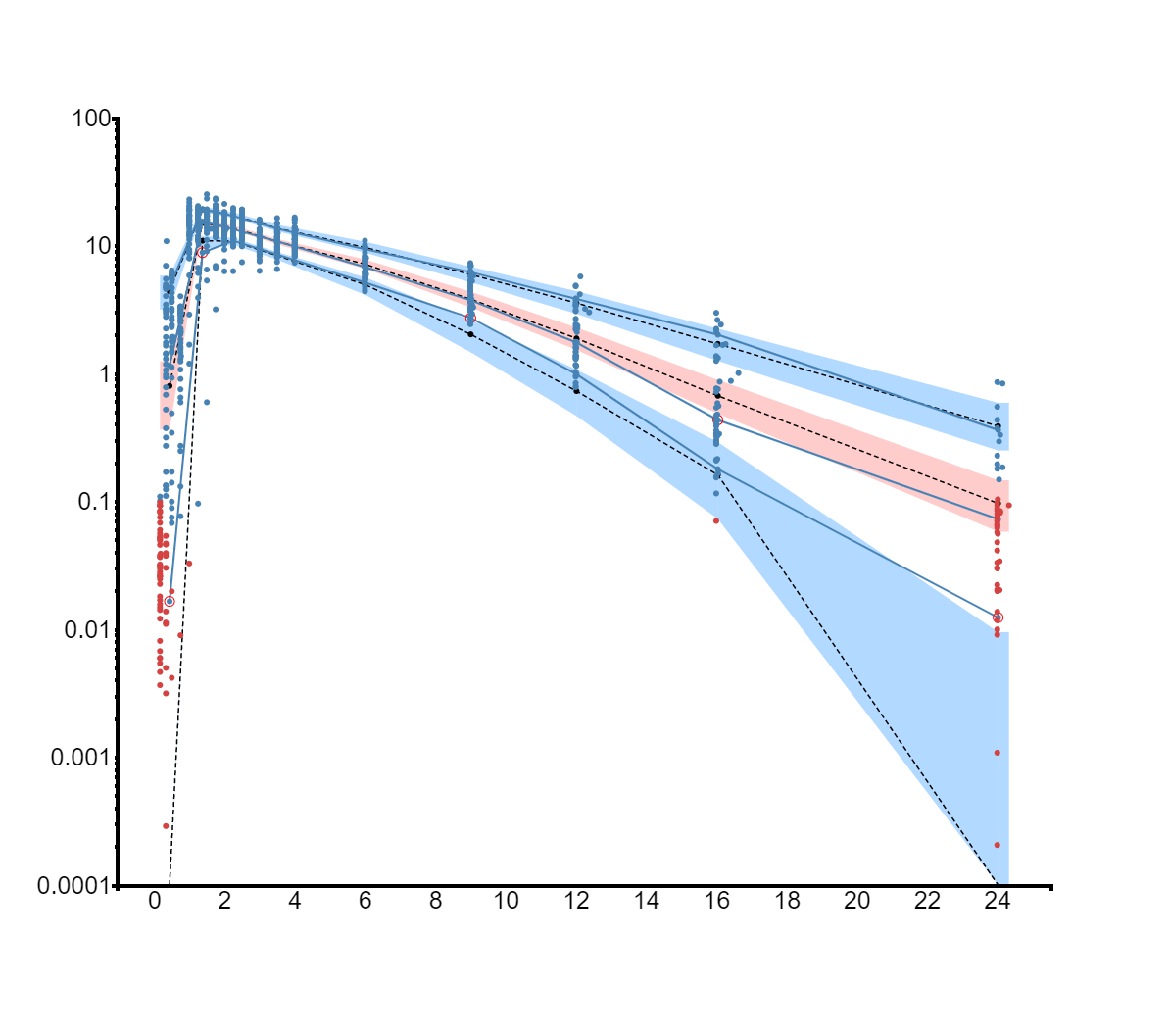


**Supplementary Fig 3.** Prediciton corrected visual predictive check (n = 1000) of [A] sex + BW and [B] FFM covariate model. Solid blue dots represent observed concentrations. Solid red dots represent BQL data. Solid blue lines represent median, 10^th,^ and 90^th^ percentiles of observed concentrations. Shaded areas are the model-predicted 90% confidence intervals of 10^th^, 50^th^, and 90^th^ percentiles (lower blue area, red area, upper blue area, respectively). Black dotted lines represent medians of the respective confidence intervals of simulated data.
